# Auditory cortex hypoperfusion: a metabolic hallmark in Beta Thalassemia

**DOI:** 10.1186/s13023-021-01969-0

**Published:** 2021-08-05

**Authors:** Renzo Manara, Sara Ponticorvo, Silverio Perrotta, Maria Rosaria Barillari, Giuseppe Costa, Davide Brotto, Rosanna Di Concilio, Angela Ciancio, Elisa De Michele, Pasquale Alessandro Carafa, Antonietta Canna, Andrea Gerardo Russo, Donato Troisi, Martina Caiazza, Federica Ammendola, Domenico Roberti, Claudia Santoro, Stefania Picariello, Maria Sole Valentino, Emanuela Inserra, Roberta Carfora, Mario Cirillo, Simona Raimo, Gabriella Santangelo, Francesco di Salle, Fabrizio Esposito, Immacolata Tartaglione

**Affiliations:** 1 Neuroradiology, Department of Neuroscience, University of Padova, Padua, Italy; 2 Dipartimento di Medicina e Chirurgia, Scuola Medica Salernitana, Università di Salerno, Fisciano, Italy; 3 Dipartimento della Donna, del Bambino e della Chirurgia Generale e Specialistica, Università degli Studi della Campania “Luigi Vanvitelli”, Via L. De Crecchio 4, 80138 Naples, Italy; 4 Università degli Studi della Campania, Naples, Italy; 5 Università di Padova, Padua, Italy; 6 Dipartimento di Pediatria, Ospedale “Umberto I”, Nocera Inferiore, Italy; 7 Unità Operativa Ematologia - Day Hospital di Talassemia, Ospedale “Madonna Delle Grazie”, Matera, Italy; 8 Medicina Trasfusionale AUO “San Giovanni di Dio e Ruggi D’Aragona”, Salerno, Italy; 9 Università di Salerno, Fisciano, Italy; 10 Clinic of Child and Adolescent Neuropsychiatry, Department of Mental Health, Physical and Preventive Medicine, University of Campania “Luigi Vanvitelli”, Naples, Italy; 11 Department of Advanced Medical and Surgical Sciences, University of Campania “Luigi Vanvitelli”, Naples, Italy; 12 Department of Psychology, University of Campania ‘Luigi Vanvitelli’, Caserta, Italy

**Keywords:** Thalassemia, Hearing loss, Brain, Perfusion, Transfusion medicine

## Abstract

**Background:**

Sensorineural hearing loss in beta-thalassemia is common and it is generally associated with iron chelation therapy. However, data are scarce, especially on adult populations, and a possible involvement of the central auditory areas has not been investigated yet. We performed a multicenter cross-sectional audiological and single-center 3Tesla brain perfusion MRI study enrolling 77 transfusion-dependent/non transfusion-dependent adult patients and 56 healthy controls. Pure tone audiometry, demographics, clinical/laboratory and cognitive functioning data were recorded.

**Results:**

Half of patients (52%) presented with high-frequency hearing deficit, with overt hypoacusia (Pure Tone Average (PTA) > 25 dB) in 35%, irrespective of iron chelation or clinical phenotype. Bilateral voxel clusters of significant relative hypoperfusion were found in the auditory cortex of beta-thalassemia patients, regardless of clinical phenotype. In controls and transfusion-dependent (but not in non-transfusion-dependent) patients, the relative auditory cortex perfusion values increased linearly with age (*p* < 0.04). Relative auditory cortex perfusion values showed a significant U-shaped correlation with PTA values among hearing loss patients, and a linear correlation with the full scale intelligence quotient (right side *p* = 0.01, left side *p* = 0.02) with its domain related to communication skills (right side *p* = 0.04, left side *p* = 0.07) in controls but not in beta-thalassemia patients. Audiometric test results did not correlate to cognitive test scores in any subgroup.

**Conclusions:**

In conclusion, primary auditory cortex perfusion changes are a metabolic hallmark of adult beta-thalassemia, thus suggesting complex remodeling of the hearing function, that occurs regardless of chelation therapy and before clinically manifest hearing loss. The cognitive impact of perfusion changes is intriguing but requires further investigations.

**Supplementary Information:**

The online version contains supplementary material available at 10.1186/s13023-021-01969-0.

## Background

Beta-thalassemia is an inherited blood disorder caused by defective production in the beta chain of hemoglobin. The most severe forms require regular blood transfusions to survive (Transfusion-Dependent Thalassemia, TDT) exacerbating multisystem iron overload. Overall survival has strikingly changed after the introduction of iron chelators that enabled the prevention and management of iron related systemic complications [1]. However, prolonged chelation therapy with the first chelator (deferoxamine) brought to light an outbreak of sensorineural hearing loss in surviving patients and it provided evidence of dose related ototoxicity, for which a correlation with the dose (therapeutic index: dosage/ferritin) was suggested [2]. This led to routine auditory assessments, especially in TDT patients, and to a more tailored treatment management for preventing iatrogenic hearing deficits [3]. Nonetheless, a recent meta-analysis still showed hearing deficits in nearly one fourth of pediatric beta-thalassemia patients treated with deferoxamine [4]. The latter observation challenged the role of deferoxamine in the pathogenesis of hypoacusia in beta-thalassemia and raised the suspicion that other causative factors could be equally, or even more, important. In addition, hearing loss has been reported also in patients undergoing iron chelation with deferiprone and deferasirox [5–7], the most recent and oral iron chelators. All these data brought out how our knowledge on hearing deficits in beta-thalassemia is far too little, especially while dealing with adult populations, non-Transfusion-Dependent Thalassemia (NTDT), other chelators than deferoxamine, or even patients naive for iron chelation [8]. Most importantly, an important issue of hearing deficit has been so far completely neglected in beta-thalassemia, i.e. the possible involvement of the brain in terms, for example, of auditory cortex metabolism or possible relationships with cognitive functioning. Indeed, hearing loss has been found to be associated with significant auditory cortex perfusion changes in age-related hearing loss [9], providing intriguing evidence about the interactive link between sensory deficit and brain metabolism. On the other hand, hearing loss might favor social isolation, depression, and cognitive decline [10], that indeed have been repeatedly shown in beta-thalassemia [11–13]. in spite of no evidence of brain iron overload or increased parenchymal vascular-like lesion burden [14–16]. Intriguingly, verbal abilities seem to be the most severely affected both in TDT and NTDT patients [13].

In this scenario, there is a growing need to better define the hearing loss features, including type, prevalence, pathogenesis, evolution, and impact on cognitive functioning. This effort would be important for disentangling the possible pathogenic links and compensatory mechanisms, to identify new targets for prevention and management of some of the disease related aspects that might considerably impact on patients’ quality of life. We performed a multicenter single-scanner cross-sectional MRI study analyzing non-invasively in beta-thalassemia patients and healthy controls the auditory cortex perfusion changes occurring in relation with clinical phenotype, age, audiological findings and cognitive performances.

## Methods

### Subjects

Study subjects were patients with transfusion-dependent and non-transfusion-dependent Thalassemia (TDT and NTDT, respectively) aged more than 16 years and healthy controls who had participated in previous multimodal brain MRI and neurocognitive studies [13, 14]. The study was approved by the Ethic Committee (approval number 161/15), and all subjects signed an Informed Consent before data collection.

### Audiological evaluation

Most patients were undergoing annually pure tone audiometry as per clinical practice (see online material); the evaluations closest to the MRI assessments were collected; for those patients who did not have an audiological test available (i.e. NTDT patients naive for ICT), a pure-tone audiometry was purposefully performed.

Pure Tone Average (PTA) was calculated as the mean in dB of the four central frequencies tested in the tonal audiometric examination (500,1000,2000,4000 Hz). Normal hearing was defined if PTA was between 0 and 25 decibels (dB nHL); the degree of hearing loss was classified according to World Health Organization—Grades of Hearing Impairment in different levels of severity: mild hearing loss (26–40 dB nHL), moderate hearing loss (41–60 dB nHL), severe hearing loss (61–80 dB nHL) and profound hearing loss (> 81 dB nHL).

Additionally, three main groups were identified based on audiometric profile: patients with normal audition (NA, PTA ≤ 25 dB), patients with hearing loss in at least one frequency but normal PTA (fHL), and patients with overt hearing loss (HL, PTA > 25 dB).

### MRI data evaluation

All study participants underwent brain MRI on the same 3.0 T scanner (MAGNETOM Skyra, Siemens, Erlangen Germany) with a 20-channel head and neck coil.

Cerebral Perfusion weighted maps (PWI) were obtained from brain images acquired using a 3D Pulsed Arterial Spin Labeling (PASL) sequence with labeling scheme FAIR Q2TIPS, TR = 5000 ms, TE = 16.38 ms, matrix-size 64 × 64 voxels, voxel-resolution = 3 × 3 × 3 mm^3^, bolus-duration 700 ms, inversion-time (TI) = 1990 ms, 2 repetitions, 50 slices, (total acquisition-time: 5.25 min). This series was acquired with the subject at rest with eyes open. For anatomical reference a 3D-T1-weighted magnetization prepared rapid gradient echo (MPRAGE) sequence was also acquired with TR = 2400 ms, TE = 2.25 ms, resolution = 1 × 1 × 1 mm^3^, matrix-size = 256 × 256, generalized autocalibrating partially parallel acquisitions (GRAPPAs) factor of 2 in phase-encoding direction.

The perfusion analysis method is reported in the online material.

Since the interest was focused regionally on the auditory cortex, to reduce the intrinsic inter-subject variability related to both physiological and technical aspects of ASL acquisition, relative cerebral blood flow (rCBF) values were obtained after normalization to the mean regional rCBF value of a non-auditory sensory brain region. For this purpose the mask of bilateral occipital cortex was obtained using pickatlas toolbox in SPM [17] and the rCBF mean value was extracted for each subject and used as a normalization factor. For the voxel-wise analyses a general linear model (GLM) full factorial design (as implemented in SPM) was used with one between subjects factor (group) of three levels (healthy controls, TDT, NTDT) and one covariate (age). The voxel-based analysis was carried out locally in the bilateral auditory cortex defined by an anatomical mask according to a cytoarchitectonic atlas [18]. T-maps were thresholded at *p* < 0.001 voxel-level and only clusters at *p* < 0.05 family-wise error corrected at the cluster level were considered significant.

### Cognitive functioning assessment

Study subjects underwent a pool of psychometric tests included in the Wechsler Adult Intelligence Scale-Fourth Edition (WAIS-IV) [13]. For this analysis Full-Scale Intelligence Quotient (FSIQ) and WAIS main domain scores were considered (see online material).

### Statistical analysis

Comparisons between groups were performed using the T test, the Mann–Whitney U test and the Chi-square test (or the Fisher Exact test when required) for respectively normally distributed, ordinal and qualitative non ordinal variables. The linear correlation between two variables was tested using Pearson's correlation. Statistical significance was set at *p* < 0.05. Finally, an analysis of covariance (ANCOVA) was used to assess the linear dependence of the mean rCBF values in the AC and the PTA separately for the groups with hearing impairment (HL and fHL). Only descriptively, the same dependence was also investigated using a polynomial fitting (bsquare fit).

## Results

### Subjects

Seventy-seven patients (54 TDT and 23 NTDT) and 56 healthy controls were enrolled; demographics and clinical characteristics are shown in Table 1. Beta-thalassemia patients did not differ from controls for age and gender, though TDT were older than NTDT patients (*p* < 0.01).

**Table 1:** Patients’s demographics and clinical characteristics; *mean Hb in TDT refers to pre transfusional values; LIC: liver iron concentration; DFO: Deferoxamine; DFP: Deferiprone; DFX: Deferasirox

	TDT (n = 54)	NTDT (n = 23)	All Patients (n = 77)	Controls (n = 56)
mean age, years	36.4 ± 9.2	30.3 ± 11.5	34.6 ± 10.5	33.9 ± 10.8
Females, n(%)	34 (62.9)	13 (56%)	47 (61.0)	36 (64.3)
Splenectomized, n (%)	35 (64.8)	9 (39%)	44 (57.1)	0
Hb*(g/dl), mean ± SD	9.2 ± 0.5	9.3 ± 0.9	9.3 ± 0.8	NA
Hb at MRI (g/dl), mean ± SD	9.6 ± 1.1	9.3 ± 0.9	9.5 ± 1	NA
Ferritin (ng/ml), mean ± SD	877.0 ± 684.4	364.1 ± 290.8	736.2 ± 641.7	NA
LIC (mg/gdw), mean ± SD	4.3 ± 3.0	6.7 ± 5.4	4.9 ± 3.7	NA
Iron Chelation Therapy				
DFO ever, n (%)	50 (92.6)	6 (26.1)	56 (72.7)	0
ongoing, n (%)	14 (25.9)	2 (8.7)	16 (20.8)	0
current dose, mg/kg	35.8 ± 3.0	20.0 ± 1.6	33.5 ± 6.4	0
DFP ever, n (%)	21 (38.9)	0	21 (27.3)	0
ongoing, n (%)	4 (7.4)	0	5 (6.5)	0
current dose, mg/kg	73.6 ± 2.4	0	73.6 ± 2.4	0
DFX ever, n (%)	50 (92.6)	8 (34.8)	58 (75.3)	0
ongoing, n (%)	35(64.8)	4 (17.4)	38 (49.3)	0
current dose, mg/kg	27.8 ± 8.1	10.1 ± 0.6	21.5 ± 10.4	0

### Pure tone audiometry

**Fig. 1:**
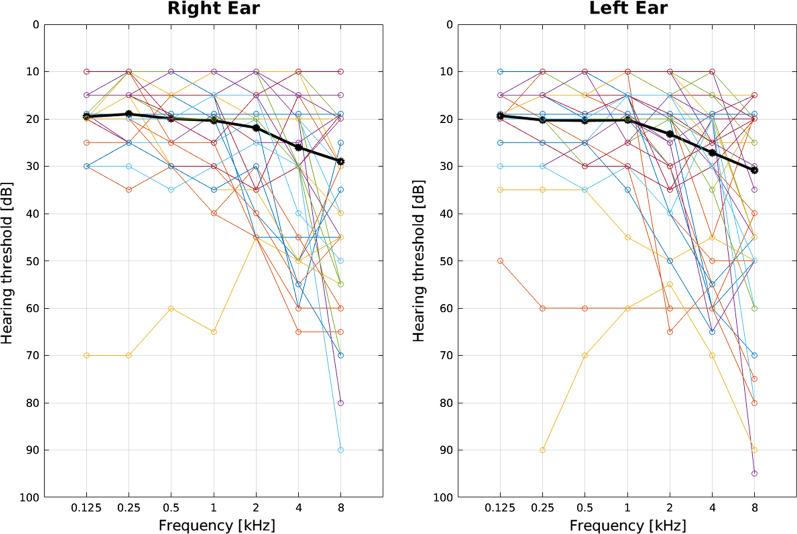
Audiometric thresholds for air conduction in the pure tone audiometry for all the patients (n = 71) and the two ears. Black lines indicate mean hearing loss across patients for each tested frequency

Seventy-one patients (51 TDT and 20 NTDT, mean-age 34.3 ± 10.6 years, 44 females) underwent auditory assessment; air conduction charts are presented in Fig. 1.

Any degree of hearing deficit was noted in 37/71 patients (52.1%, 19/37 females; 30/51 TDT, 7/20 NTDT, *p* > 0.05). Mean-age of patients with hearing deficit was 37.8 ± 9.5 versus normal hearing patients 30.5 ± 10.5 (*p* = 0.003). Three out of seven NTDT patients with abnormal audiometry never took iron chelation therapy. Overt hypoacusia (PTA > 25 dB) was found in 25/71 patients (35.2%; 19 TDT and 6 NTDT) and 12/71 (14.9%; 11 TDT and 1 NTDT) were classified as fHL (one or more abnormal frequencies in spite of PTA < 25 dB). Among patients with PTA > 25 dB, hypoacusia was mild in 18/25 (mean PTA right 28.4, range 15–40; left 29.6, range 17.3–40), moderate in 5/25 and severe in 2/25 (ipsilaterally to a cholesteatoma and otosclerosis respectively).

In 28/37 patients hearing loss was sensorineural (SNHL, including both fHL and HL; five were NTDT patients): it was bilateral and symmetric in 50% of the subjects. Conductive deficits were found in 3/37 (two because of otosclerosis and one because of unilateral cholesteatoma); three a mixed deficit, one had SNHL at one side and conductive at the other, two had SNHL one side and mixed the other.

### MRI analyses: regional relative brain perfusion

Whole brain perfusion is expected to be globally increased due to anemia [19] and this was clearly seen when comparing beta-thalassemia patients and controls (data not shown). To overcome this limit we calculated a rCBF ratio (between two non-overlapping regions both involved in stimuli perception, namely the auditory and the visual cortices) thus minimizing the effect of hemoglobin levels on the cerebral perfusion. This ratio also allowed us to consider our pulsed arterial spin labelling (PASL) sequence suitable for quantification of cerebral blood flow providing meaningful and comparable measures of rCBF.

**Fig. 2:**
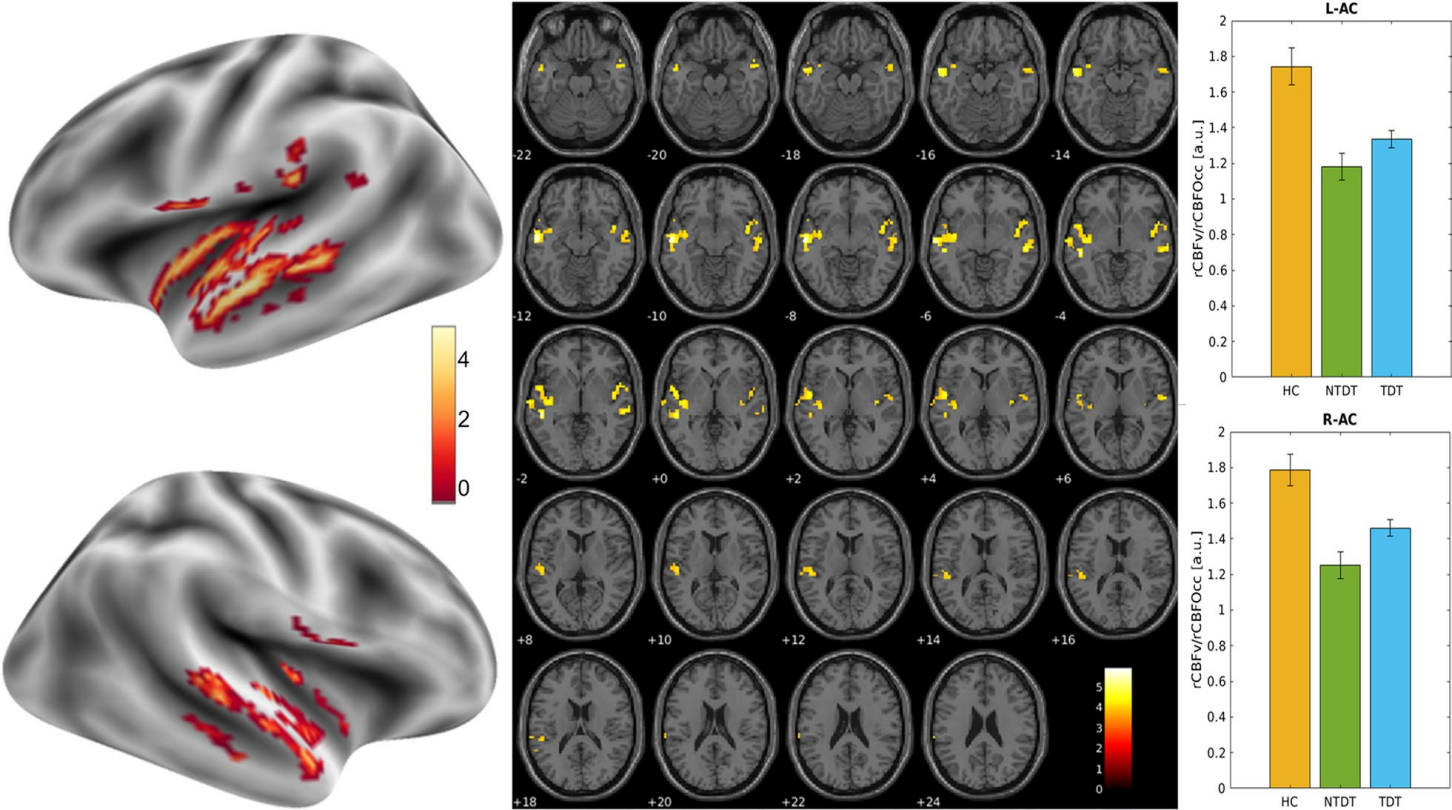
**Left:** T-maps of the statistical comparison between Healthy Controls and Thalassemic patients (TDT and NTDT) showed respectively on inflated brain surfaces and on the single subject T1w anatomical image. **Right:** Bar plot (and standard deviation bars) of the mean perfusion values across the three groups in the clusters of significant differences. Patients had significantly lower perfusion compared to Healthy Controls in both left (L-AC) and right (R-AC) auditory cortex. By subgroup analysis, the difference persisted significant between healthy controls and TDT; perfusion values did not differ between NTDT and TDT. rCBFv: relative cerebral blood flow values in the auditory cortex; rCBFOcc: relative cerebral blood flow in the occipital cortex

Accordingly, the auditory cortex in beta-thalassemia patients showed a significant relative hypoperfusion in the Heschl’s gyrus and the neighboring cortex bilaterally (left cluster size = 331 voxels; right clusters size = 94 voxels and 78 voxels; 1 voxel = 27mm3; Fig. 2). No differences were found between TDT and NTDT or according to chelation therapy.

**Fig. 3:**
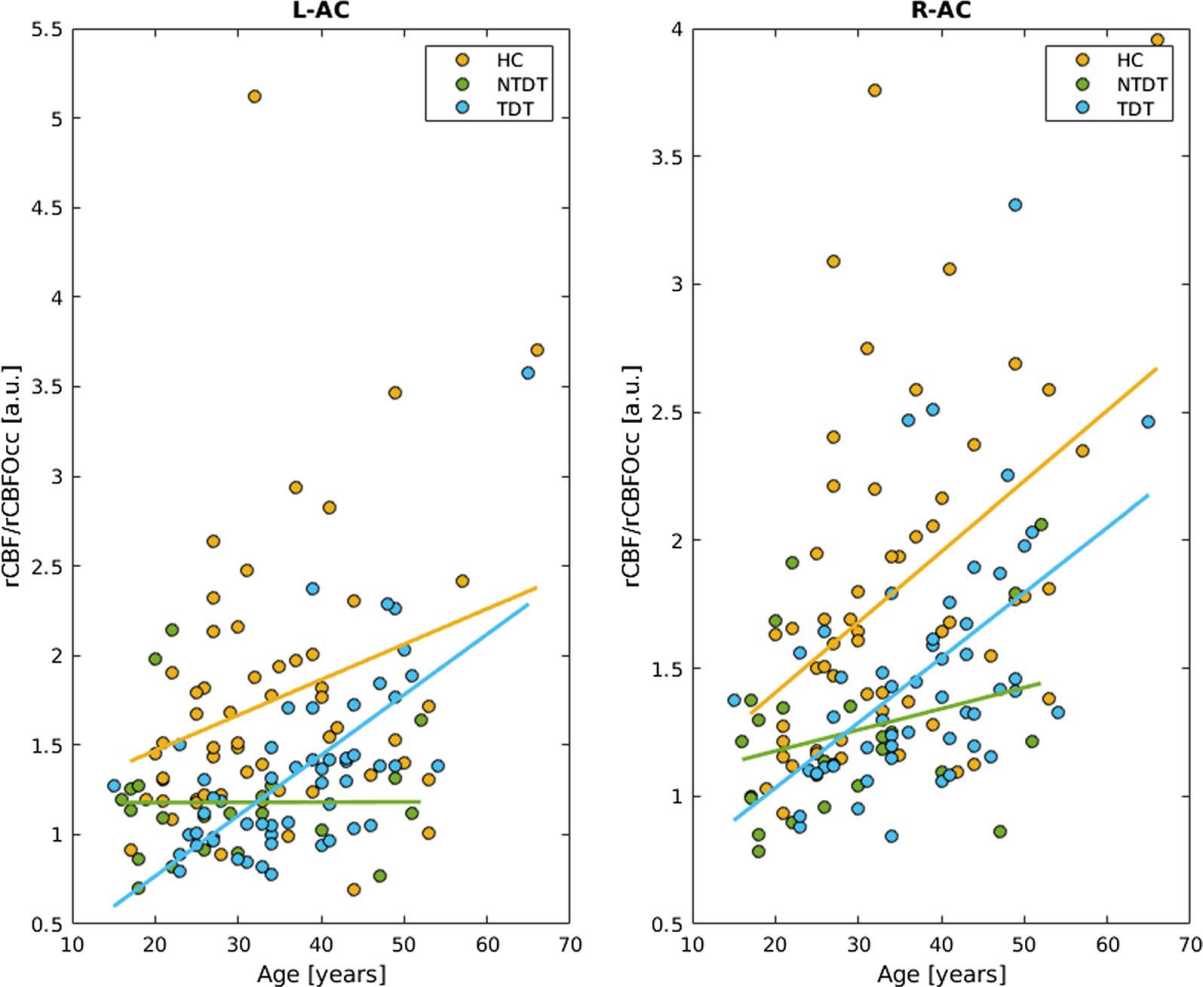
Relative left and right auditory cortex perfusion values according to age in healthy controls, Transfusion-Dependent Thalassemia (TDT) and Non-Transfusion-Dependent Thalassemia (NTDT) patients

In healthy controls, the relative left and right auditory cortex perfusion values showed a linear increase with age (left: *p* = 0.035, r = 0.28, right: *p* < 0.001, r = 0.45), that was replicated in TDT patients (left: *p* < 0.001, r = 0.67, right: *p* < 0.001, r = 0.53); in NTDT patients no significant changes appeared in the auditory cortex perfusion values with increasing age (left: *p* > 0.05, r = 0.004, right: p > 0.05, r = 0.29). (Fig. 3).

Restricting the analysis to patients with audiological characterization (n = 71), relative hypoperfusion was confirmed compared to healthy controls (left cluster size = 722 voxels and right cluster size = 264 voxels).

Among patients, after regression on age, there was no significant difference in regional perfusion values among patients with normal audition, fHL and HL (Additional file 1: Fig. S1﻿).

**Fig. 4:**
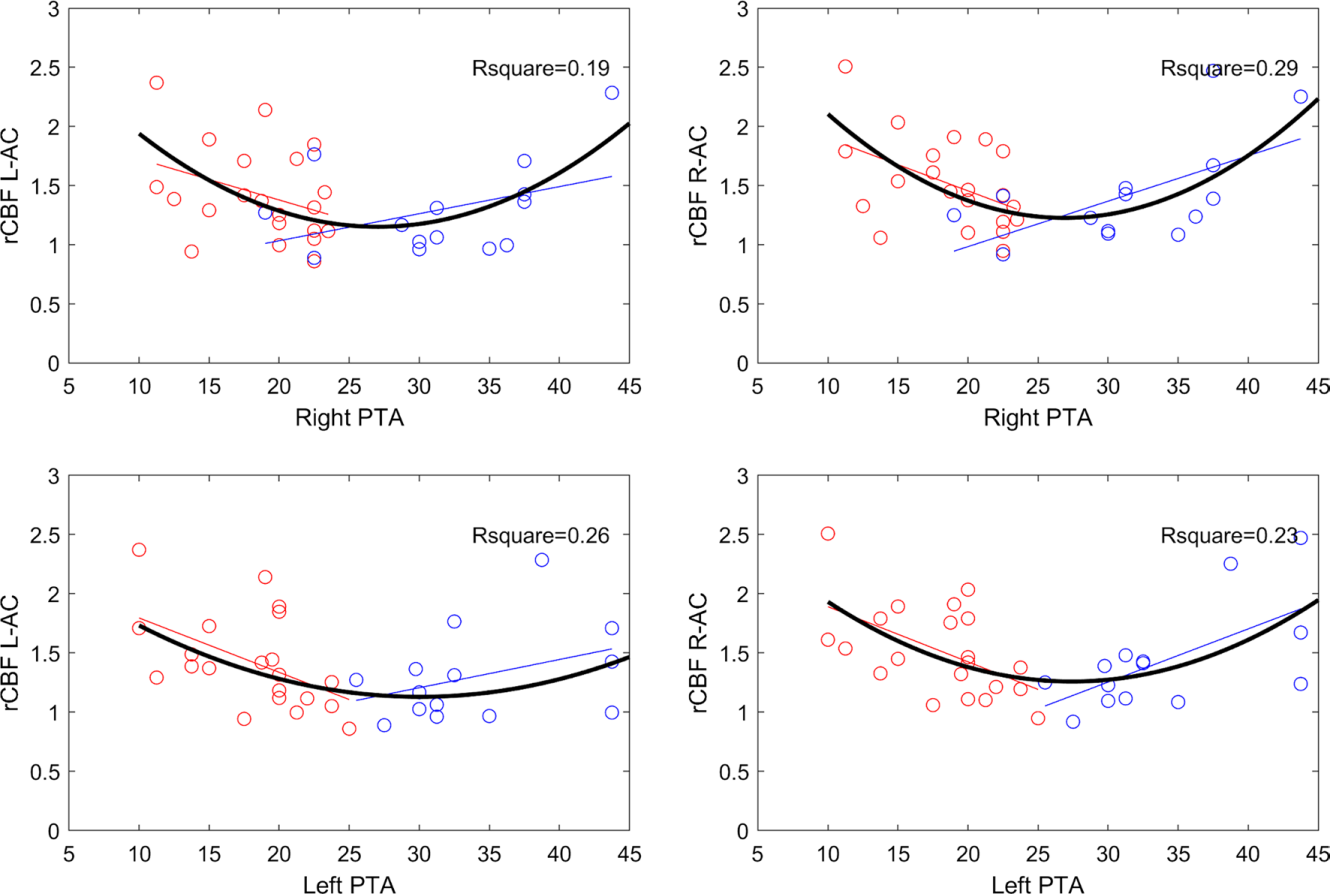
Polynomial fitting of the second order between relative left and right auditory cortex perfusion values (rCBF L-AC and rCBF R-AC) and Pure Tone Average (PTA). Circles and lines indicate the subjects and the linear relationship between PTA and rCBF (red for hearing loss in at least one frequency (fHL), blue for hearing loss with abnormal Pure Tone Average (HL)); the curve in black is the second-order polynomial fitting (Robust fit bisquare) and the relative R-square value considering all the subjects together. Three subjects were excluded from this analysis because of outlier values of PTA, secondary to cholesteatoma in 1 and otosclerosis in 2

However, PTA of the two hearing loss groups (fHL and HL), taken separately, correlated with rCBF values. At first, a linear regression was performed accounting for the groups (using an analysis of covariance model), that revealed a significant interaction effect (HL-group × PTA) on the rCBF values. Observing the different behavior of the two groups the correlation was further described with a second-order polynomial fitting using the curve fitting toolbox of MATLAB (Fig. 4).

**Fig. 5:**
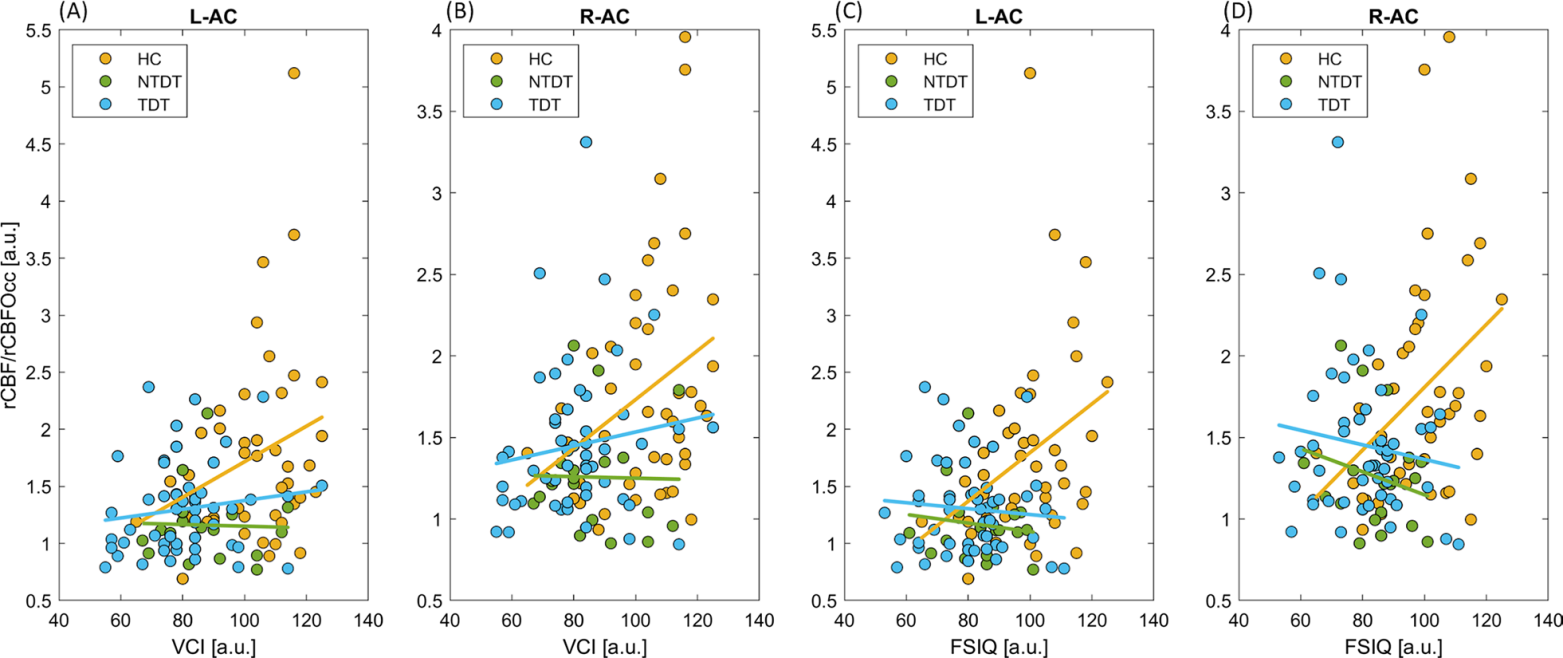
Relative left and right auditory cortex perfusion values according to Verbal Comprehension Index (VCI) (**A**, **B**) and Full Scale Intelligence Quotient (FSIQ) (**C**, **D**) in Healthy Controls (HC), Transfusion-Dependent Thalassemia (TDT) and Non-Transfusion-Dependent Thalassemia (NTDT) patients

In controls but not in beta-thalassemia patients, the auditory/visual cortex perfusion ratio directly correlated with the FSIQ score (right side *p* = 0.01, left side *p* = 0.02) and with the cognitive domains primarily involved in communication skills (Fig. 5). Correlations with all WAIS domains are shown in Table 2 and the e-Table in Additional file 1.

**Table 2:**
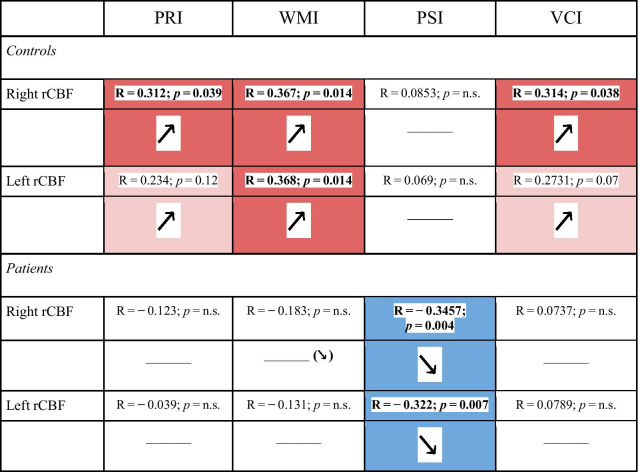
Correlation of MRI cerebral blood flow and cognitive scores.

Pearson’s correlations between cognitive scores derived by the WAIS and right and left auditory/visual cerebral area perfusion ratio in healthy controls and patients. *PRI* Perceptual Reasoning Index, *WMI* Working Memory Index, *PSI* Processing Speed Index, *VCI* Verbal Comprehension Index. legend: red cells with upwards arrow = positive association with R > 0.2; pink cells with upwards arrow = trend of positive association with R > 0.2; blue cells with downwards arrow = negative association with R < − 0.2; ^____^ no association (− 0.2 < R < 0.2); significant *p* values are reported in bold

No correlation was found in the patients' group between cognitive functioning scores and audiometric profile (FSIQ *p* = 0.46, VCI *p* = 0.3).

## Discussion

The present perfusion MRI study aimed at characterizing brain perfusion changes of the auditory cortex on a large sample of beta-thalassemia patients. According to our findings, bilateral relative hypoperfusion of the auditory cortex seems to represent an early hallmark of this hemoglobinopathy, largely independent of the clinical phenotype and hypoacusis condition. However, some aspects have to be considered to better understand the complex metabolic and functional auditory cortex changes that characterize both healthy and beta-thalassemia subjects.

### Hearing loss in beta-thalassemia patients

Hearing loss is common in beta-thalassemia, especially among patients treated with iron chelators [5], and it is rather invalidating as not only it affects communicative skills,, but it might also impact on cognitive functioning [10]. In a recent meta-analysis, hearing deficit has been found in more than 27% of pediatric beta-thalassemia patients treated with deferoxamine. Surprisingly, sensorineural hearing loss (i.e. the form attributed to chelation therapy) did not result as the sole or the main form of hearing deficit. In fact, it was found in 10.6% of treated patients, while 9.1% had conductive and 14.9% had mixed hearing loss, thus challenging the pathogenic link between hearing loss and chelation treatment, at least in the pediatric age [4].

In our sample of adult beta-thalassemia patients, hearing deficit was found in about half of cases and about one third had PTA impairment consistent with mild-to-moderate hypoacusia. Even though a precise characterization of hypoacusia in beta-thalassemia is beyond the scope of this study, our findings underly that (1) most likely hearing loss worsens and becomes more frequent with aging, as hypocusic patients’ mean-age was 8 years higher than what observed in normal hearing patients; (2) differently from what has been reported for the pediatric age, in adulthood most patients present with sensorineural hearing loss, and (3) even patients naive for iron chelation therapy may present with sensorineural hearing impairment.

### Auditory cortex perfusion and beta-thalassemia phenotype

The main novel result of the present study is the robust evidence of decreased relative perfusion of auditory cortex in beta-thalassemia. This finding takes into account (and is therefore not affected by) the fact that the global (whole brain) perfusion was expectedly increased in beta-thalassemia as a (known) compensatory mechanism to lower mean hemoglobin levels.

To overcome the confound of global hyperperfusion, a ratio between the rCBF in each brain location and the rCBF in the occipital cortex was first obtained, assuming that the optic pathway and, more generally, the visual function are not affected in this condition (similar findings were found also considering the global whole brain perfusion as a reference, data not shown). Interestingly, the relative hypoperfusion of the auditory cortex was observed in both clinical phenotypes (TDT and NTDT), thus highlighting that this phenomenon does not depend on disease severity and affects all forms of beta-thalassemia regardless of transfusion dependency. In addition, the ratio between auditory and visual cortex perfusion values increases with age in healthy controls in line with what observed by Hugenschmidt et al. [20] In their study (so far, the sole investigating age-related normal auditory cortex perfusion changes), they showed in older people greater auditory activity than in younger adults to background auditory stimuli [20].

Accordingly, TDT patients showed a similar trend of increasing relative auditory cortex perfusion, although this trend remained at any age below what was observed in healthy controls.

In contrast, NTDT patients showed perfusion values close to healthy controls at a young age. However, relative perfusion values remain substantially constant with increasing age, so that during adulthood (i.e. starting at age 40) they become even lower than those of TDT patients.

If confirmed on larger age-ranges samples, our findings reveal a different time course of perfusion among patients treated conservatively without blood transfusions, that strikingly differs from what observed in normal controls.

In this case, the pathogenic link between age related perfusion changes and NTDT related features (e.g. chronically lower mean hemoglobin levels, increased hypercoagulability state, higher percentage of pathological red blood cells) would then need to be further explored.

### Auditory cortex perfusion and audiometric profile in beta-thalassemia patients

Hypoperfusion in the auditory cortex did not correlate with the audiological phenotype as patient subgroups with normal hearing, fHL and HL did not differ significantly in terms of mean relative perfusion values. However, auditory cortex perfusion values were intriguingly linked with the hearing loss severity in the patient subgroups with auditory deficits.

These apparently contradictory findings (i.e. no perfusion differences among beta-thalassemia hearing loss subgroups together with a significant correlation between perfusion and hearing loss severity) are partly explained by the decreasing perfusion with increasing severity in the fHL subgroup and the concomitant increasing perfusion with increasing severity in the HL subgroup. Considering hearing deficit in beta-thalassemia patients as a single phenomenon that worsens by involving both more, and more severely, the tested frequencies, the correlation between hearing deficit and perfusion is well depicted by a curve that associates the lowest perfusion levels to an intermediate hearing loss severity (i.e. a PTA close to 25 dB). These findings seem to indicate that the perfusion initially correlates with the hearing deficit, but that other compensatory mechanisms likely act whenever the deficit overcomes a PTA threshold of 25 dB. Interestingly, a compensatory increase in perfusion occurs at this threshold, at which individuals experience difficulty in understanding everyday speech [21].

Our findings among normal hearing beta-thalassemia patients prompt the counterintuitive hypothesis that auditory cortex hypoperfusion could be not secondary to hearing loss, rather it might pre-date the onset of clinically detectable hearing deficits. This hypothesis could also explain the common detection of sensorineural hearing deficit among NTDT patients naive for iron chelators.

### Auditory cortex hypoperfusion and cognitive functioning in beta-thalassemia

Normal hearing is crucial in cognitive functioning as it allows effective responses to external stimuli, contributing to comprehension, vocabulary and memory; it is therefore conceivable that the metabolic activity of the auditory cortex in healthy subjects correlates linearly with the cognitive scores such as the FSIQ. In contrast, the relative auditory cortex perfusion was found in both beta-thalassemia phenotypes persistently low, independently of cognitive functioning. The lack of increasing perfusion with increasing cognitive functioning in beta-thalassemia made the difference in perfusion vs healthy controls even larger among those with higher cognitive performances, as if the auditory cortex metabolism were not further recruitable in response to increased cognitive functional needs. Actually, the inability to tune the auditory cortex metabolic level to the cognitive functioning could be a possible, so far unexplored, explanation of the generally reduced cognitive performances observed in the beta-thalassemia population, especially in the TDT subgroup and regarding primarily the verbal-comprehension tasks.[13]. Compared to the other cognitive domains, the aberrant relationship observed between Processing Speed Index (PSI) scores and auditory cortex metabolism could be explained by the strong dependence of this domain from the visual performances, as the relative perfusion values were driven by the ratio between auditory and visual cortex perfusion. This could result in no correlation in healthy controls due to concurrent perfusion changes in both cortices, and in a negative correlation in patients due to the increase in the visual cortex not compensated by a congruous increase in the auditory cortex perfusion. In addition, in our beta-thalassemia population the PSI scores among the WAIS domains were the least affected compared to controls [13].

Two other points deserve to be further addressed. The fact that, notwithstanding the above mentioned metabolic impairment, some beta-thalassemia patients reach high level cognitive performances might suggest the existence and recruitment of alternative neural networks for compensating the low response of the auditory cortex. Indeed, the lack of correlation between hearing loss severity and cognitive functioning among beta-thalassemia patients supports the hypothesis of alternative recruitments of less efficient extra-auditory neural networks. Secondly, the more severe metabolic involvement of the left auditory cortex (the cluster size of decreased relative perfusion is nearly three times larger than the contralateral one) seems to further underline the link between the observed metabolic derangement and the cognitive language-related scores, as the left is known to be the dominant hemisphere for most language tasks.

Notably, auditory cortex hypoperfusion has been recently shown to distinguish patients with age-related hearing loss due to hair cell loss, that typically involves the high frequencies with a pure tone audiogram rather similar to what observed in beta-thalassemia patients [9]. However, in spite of audiogram similarities, these two conditions present basic though intriguing metabolic differences. In beta-thalassemia the hypoperfusion is more evident on the left auditory cortex while in age-related hypoacusia the hypoperfusion prevails on the right side [9]. Being the right hemisphere more involved in frequency discrimination, this finding seems to suggest that in beta-thalassemia the role of frequency perception is less determinant than the involvement of the semantic processes of language in real-world auditory scenarios [22].

### Auditory cortex hypoperfusion in beta-thalassemia patients: hypothesis

The phenomenon of auditory cortex hypoperfusion independent from hearing deficits is rather difficult to explain, even considering differential development paths of the regional brain cortex or regional differences in brain vasculature. It is well known, for example, that the auditory cortex is one of the few regions that maturate early during the fetal life. A regionally different timing of brain cortex maturation could result in a selective vulnerability of the early developing cortices to early ischemic and metabolic events. However, an effect of thalassemia on the auditory cortex appears unlikely during gestation or early postnatal months as fetal hemoglobin production is preserved. In addition, the visual cortex, that shares a similar maturation course during fetal brain development, does not seem to be concomitantly affected.

Analogously, the anatomic features of the intracranial vascular system do not seem to expose the auditory cortex to specific perfusion injuries. The auditory cortex does not belong to watershed territories; on the contrary, this region is well supplied by branches of the middle cerebral artery, the main cerebral artery of the developing and mature brain.

Therefore, the hypothesis of a disease-related selective regional vulnerability is intriguing but not likely. To make this issue even more puzzling, data on the pediatric brain perfusion are still lacking in beta-thalassemia and it is unknown whether the perfusion deficit of the auditory cortex appears at birth or even earlier. The present study focused on adults because of reasonable ethical concerns in investigating small children requiring sedation and because normative perfusion MRI data on the developing brain are still lacking and difficult to be achieved. Auditory cortex hypoperfusion predisposing to subsequent hearing loss is a hypothesis that could partly explain the high rate of hearing deficit in beta-thalassemia. So far, hearing loss in beta-thalassemia has been considered a peripheral sensorineural high-frequency auditory problem mostly due to chelation treatment. However, data on the link between chelation therapy at current dosages and hearing deficits are rather conflicting [2, 8, 23]. Even the analysis of the hearing deficits in our sample shows that the scenario is much more intricate: 15% of NTDT patients presented with sensorineural hearing loss in spite of no history of chelation therapy or any other known cause of hearing impairment. The fact that auditory cortex hypoperfusion is not limited within specific clinical phenotypes and is not associated with any chelation treatment, could suggest its possible role in increasing the vulnerability of hearing to treatments and comorbidities such as otitis media episodes, high serum iron levels, decreased hemoglobin levels, that partly occurs independently from chelation therapy.

## Limits

Even though this is the first study that provides a detailed picture of the metabolic status of the auditory cortex in beta-thalassemia in relation to the audiogram profile and the cognitive scores, there are limits that should be highlighted to define the possible future development in this field of research. First of all, we used a non-invasive ALS-MRI technique that does not provide absolute perfusion values. For this reason, we considered a ratio with a likely not involved brain cortex (namely the visual region) introducing possible confounders. Secondly, the present study included only a couple of dozens of NTDT patients; as they likely represent a distinct beta-thalassemia patient population (lower mean hemoglobin levels, lower mean hospitalization days, etc.) studies focusing on a larger sample of NTDT are advisable. We used the WAIS assessment for cognitive functions, that is preferred in the clinical practice, though other cognitive tests used in the research context could be more appropriate in characterizing the cognitive profile of patients. Finally, our study did not include pediatric patients leaving unsolved the timing regarding the development of the observed perfusion change in beta-thalassemia. This knowledge will help us to better define, if present, possible actions to prevent brain perfusion changes in this complex hereditary hemoglobinopathy.

## Conclusions

Bilateral auditory cortex hypometabolism seems to represent an early hallmark of beta-thalassemia regardless of the clinical phenotype and the presence of hearing loss. The complex cognitive, audiological and metabolic scenario resulting from our study appears novel, extremely intriguing and needing to be further investigated with longitudinal studies. The latters will help to disentangle the pathogenic link between auditory cortex perfusion and cognitive and audiological impairment that characterize brain metabolism and function in beta-thalassemia.

## Supplementary Information

**Additional file 1**. Audiological, Cognitive functioning, MRI data evaluation.** S. Fig.1**. Relative auditory cortex perfusion values among patients with auditory evaluation.** e-Table**. Pearson’s correlations between cognitive scores derived by the WAIS and right and left auditory/visual cerebral area perfusion ratio in healthy controls and patients.

## Data Availability

Original data will be available upon request at the corresponding author.
