# Supplementary material for: Auditory cortex hypoperfusion: a metabolic hallmark in Beta Thalassemia
Source: Orphanet J Rare Dis. 2021 Aug 5;16:349. doi: 10.1186/s13023-021-01969-0 (PMC8340544; doi:10.1186/s13023-021-01969-0)
Supplement: Supplementary file 1 — Additional file 1. Audiological, Cognitive functioning, MRI data evaluation. S. Fig.1. Relative auditory cortex perfusion values among patients with auditory evaluation. e-Table. Pearson’s correlations between cognitive scores derived by the WAIS and right and left auditory/visual cerebral area perfusion ratio in healthy controls and patients. [file 13023_2021_1969_MOESM1_ESM.docx]

**Auditory cortex hypoperfusion: a metabolic hallmark in Beta Thalassemia.**

*Audiological evaluation*

At each site, all the tests were performed in a silent booth by using a standard two channel clinical audiometer (Resonance r27a). Air conduction pure tone threshold was tested for the right and left ear separately with calibrated headphones at 250 Hz - 500 Hz - 1 kHz - 2 kHz - 4 kHz - 8 kHz, while bone conduction threshold was assessed by using the bone stimulator on the mastoid bone at 250 Hz - 500 Hz - 1 kHz - 2 kHz - 4 kHz. The audiometer and headphones were calibrated to ISO (International Organization for Standardization) standards (Standardization ISO Acoustics: reference zero for the calibration of audiometric equipment Part 1, Reference equivalent threshold sound pressure levels for pure tone and supra-aural earphones, 2004.)

Threshold levels were determined with a 10dB-down/5dB-up approach [(Carhart and Jerger 1959)](https://paperpile.com/c/uLeDeI/i5BI). Clinical masking was used if necessary or warranted by the clinical audiologic data; otoscopic examination was always performed before the Pure Tone Audiometry. The readings recorded were plotted on the audiogram chart. The findings were reviewed and included in the study only if considered reliable by the audiologist.

*Cognitive functioning evaluation*

Study subjects underwent the Wechsler Adult Intelligence Scale— Fourth Edition (WAIS-IV).[(Tartaglione et al. 2019)](https://paperpile.com/c/uLeDeI/UNQzT) It is composed by ten main subtests that measure four cognitive indices: verbal comprehension (including similarities, vocabulary, and information subtests), perceptual reasoning (including block design, matrix reasoning, and visual puzzles subtests), working memory (digit span, and arithmetic subtests) and processing speed (symbol Search, and coding subtest). The score obtained to each of these cognitive indices was summarized in the Full-Scale Intelligence Quotient (FSIQ) that reflects the global cognitive functioning.

*MRI data evaluation*

**Sequence parameters: labeling scheme FAIR Q2TIPS, TR=5000ms, TE=16.38ms, matrix-size 64x64 voxels, voxel-resolution=3x3x3 mm^3^, bolus-duration 700ms, inversion-time (TI)=1990ms, 2 repetitions, 50 slices, (total acquisition-time: 5.25min)**

**Acquisitions were done with the subject at rest with eyes open. For anatomical reference a 3D-T1-weighted magnetization prepared rapid gradient echo (MPRAGE) sequence was also acquired with TR=2400ms, TE=2.25ms, resolution=1×1×1mm^3^, matrix-size=256×256, generalized autocalibrating partially parallel acquisitions (GRAPPAs) factor of 2 in phase-encoding direction.**

Single-subject whole-brain relative CBF (rCBF) maps were calculated from perfusion-weighted (PWI) images (i.e. control-label differences from the 3D-PASL raw image series) according to the consensus formula [(Alsop et al. 2015)](https://paperpile.com/c/uLeDeI/zAxM), which is implemented in the inline MR scanner software. Because no M0 image is acquired the rCBF maps should be considered semi-quantitative. For the group-level analysis, the single-subject rCBF maps were spatially normalized using the SPM12 toolbox (www.fil.ion.ucl.ac.uk/spm/) running on MATLAB R2017a (The MathWorks, Inc., Natick, Massachusetts, United States, www.mathworks.com) to the Montreal Neurological Institute (MNI) standard template[(Evans et al., n.d.)](https://paperpile.com/c/uLeDeI/vPWz) using a three-step procedure: first, the control images from the 3D-PASL series were averaged to obtain an image with enhanced contrast, which was aligned in each subject to the corresponding anatomical 3D-T1w image with an affine transformation. Then, all T1w images were segmented and normalized into a study specific template space with the non-linear diffeomorphic DARTEL approach [(Ashburner 2007)](https://paperpile.com/c/uLeDeI/4Iey)and then transformed to the standard MNI space. Last, the initial affine transformation, the DARTEL estimated non-linear deformation fields and an isotropic 6-mm full width at half maximum (FWHM) Gaussian kernel were applied to the rCBF maps of each subject.

To focus the analysis on the bilateral auditory cortex an anatomical mask was defined on the MNI template according to a cytoarchitectonic atlas of the auditory cortex.[(Kim et al. 2000a)](https://paperpile.com/c/uLeDeI/gUjLn)

**The mask of bilateral occipital cortex was obtained using pickatlas toolbox in SPM**[(Maldjian et al. 2003)](https://paperpile.com/c/uLeDeI/BEpo) **and the rCBF mean value was extracted for each subject and used as a normalization factor. For the voxel-wise analyses a general linear model (GLM) full factorial design (as implemented in SPM) was used with one between subjects factor (group) of three levels (healthy controls, TDT, NTDT) and one covariate (age). The voxel-based analysis was carried out locally in the bilateral auditory cortex defined by an anatomical mask according to a cytoarchitectonic atlas.**[(Kim et al. 2000b)](https://paperpile.com/c/uLeDeI/gEyu) **T-maps were thresholded at p<0.001 voxel-level and only clusters at p<0.05 family-wise error corrected at the cluster level were considered significant.**


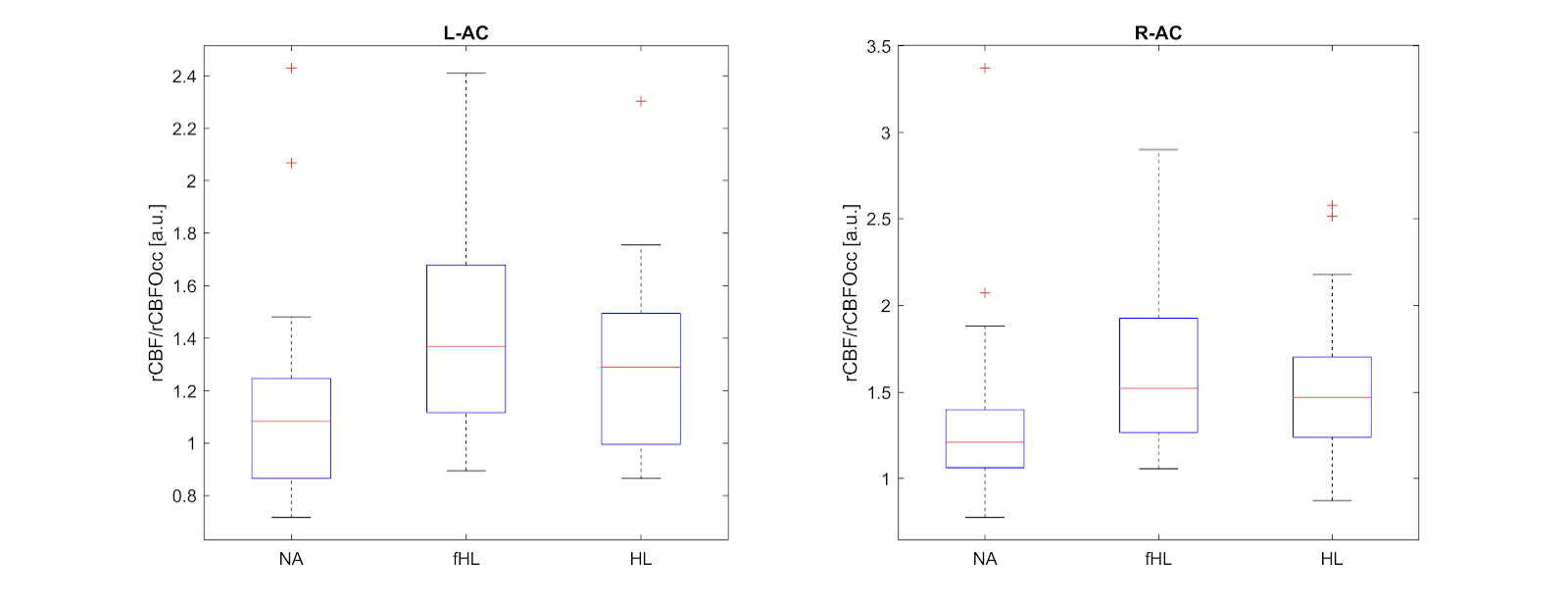


S. Fig.1: Relative auditory cortex perfusion values among patients with auditory evaluation, subdivided in Normal Audition (NA), hearing loss in at least one frequency (fHL), hearing loss with abnormal Pure Tone Average (HL). No significant difference was found among subgroups.

|  | **PRI** | **WMI** | **PSI** | **VCI** |
| --- | --- | --- | --- | --- |
| **CONTROLS** | | | | |
| Right | **R=0.312; p=0.039** | **R=0.367; p=0.014** | R=0.085; p=0.582 | **R=0.314; p=0.038** |
| Left | R=0.234; p=0.126 | **R=0.368; p=0.014** | R=0.069; p=0.656 | R=0.273; p=0.073 |
| **NTDT PATIENTS** | | | | |
| Right | R=-0.116; p=0.639 | R=-0.122; p=0.625 | R=-0.304; p=0.212 | R=-0.019; p=0.938 |
| Left | R=-0.1; p=0.684 | R=-0.111; p=0.651 | R=-0.082; p=0.739 | R=-0.033; p=0.897 |
| **TDT PATIENTS** | | | | |
| Right | R=-0.111; p=0.442 | R=-0.171; p=0.235 | **R=-0.375; p=0.007** | R=0.142; p=0.326 |
| Left | R=-0.005; p=0.973 | R=-0.111; p=0.447 | **R=-0.412; p=0.003** | R=0.146; p=0.311 |
| **ALL PATIENTS** | | | | |
| Right | R=-0.123; p=0.314 | R=-0.183; p=0.132 | **R=-0.346; p=0.004** | R=0.074; p=0.547 |
| Left | R=-0.039; p=0.750 | R=-0.131; p=0.283 | **R=-0.322; p=0.007** | R=0.079; p=0.519 |

e-Table: Pearson’s correlations between cognitive scores derived by the WAIS and right and left auditory/visual cerebral area perfusion ratio in healthy controls and patients. PRI: Perceptual Reasoning Index; WMI: Working Memory Index; PSI: Processing Speed Index; VCI: Verbal Comprehension Index. legend: red cell background = positive association R>0.2; blue cell background = negative association with R<-0.2; white cell background with -0.2<R<-0.2. NTDT: Non Transfusion Dependent Thalassemia; TDT: Transfusion Dependent Thalassemia.

References:

[Alsop, David C., John A. Detre, Xavier Golay, Matthias Günther, Jeroen Hendrikse, Luis Hernandez-Garcia, Hanzhang Lu, et al. 2015. “Recommended Implementation of Arterial Spin-Labeled Perfusion MRI for Clinical Applications: A Consensus of the ISMRM Perfusion Study Group and the European Consortium for ASL in Dementia.” *Magnetic Resonance in Medicine: Official Journal of the Society of Magnetic Resonance in Medicine / Society of Magnetic Resonance in Medicine* 73 (1): 102–16.](http://paperpile.com/b/uLeDeI/zAxM)

[Ashburner, John. 2007. “A Fast Diffeomorphic Image Registration Algorithm.” *NeuroImage* 38 (1): 95–113.](http://paperpile.com/b/uLeDeI/4Iey)

[Carhart, Raymond, and James F. Jerger. 1959. “Preferred Method For Clinical Determination Of Pure-Tone Thresholds.” *Journal of Speech and Hearing Disorders*. https://doi.org/](http://paperpile.com/b/uLeDeI/i5BI)[10.1044/jshd.2404.330](http://dx.doi.org/10.1044/jshd.2404.330)[.](http://paperpile.com/b/uLeDeI/i5BI)

[Evans, A. C., D. L. Collins, S. R. Mills, E. D. Brown, R. L. Kelly, and T. M. Peters. n.d. “3D Statistical Neuroanatomical Models from 305 MRI Volumes.” In *1993 IEEE Conference Record Nuclear Science Symposium and Medical Imaging Conference*, 1813–17. IEEE.](http://paperpile.com/b/uLeDeI/vPWz)

[Kim, J. J., B. Crespo-Facorro, N. C. Andreasen, D. S. O’Leary, B. Zhang, G. Harris, and V. A. Magnotta. 2000a. “An MRI-Based Parcellation Method for the Temporal Lobe.” *NeuroImage* 11 (4): 271–88.](http://paperpile.com/b/uLeDeI/gUjLn)

[———. 2000b. “An MRI-Based Parcellation Method for the Temporal Lobe.” *NeuroImage* 11 (4): 271–88.](http://paperpile.com/b/uLeDeI/gEyu)

[Maldjian, Joseph A., Paul J. Laurienti, Robert A. Kraft, and Jonathan H. Burdette. 2003. “An Automated Method for Neuroanatomic and Cytoarchitectonic Atlas-Based Interrogation of fMRI Data Sets.” *NeuroImage* 19 (3): 1233–39.](http://paperpile.com/b/uLeDeI/BEpo)

[Tartaglione, Immacolata, Renzo Manara, Martina Caiazza, Pasquale Alessandro Carafa, Violetta Caserta, Teresa Ferrantino, Ilaria Granato, et al. 2019. “Brain Functional Impairment in Beta-Thalassaemia: The Cognitive Profile in Italian Neurologically Asymptomatic Adult Patients in Comparison to the Reported Literature.” *British Journal of Haematology* 186 (4): 592–607.](http://paperpile.com/b/uLeDeI/UNQzT)
